# Pathway analysis of nursing interns’ professional benefit perception and influencing factors: a cross-sectional study

**DOI:** 10.3389/fmed.2025.1628232

**Published:** 2025-08-13

**Authors:** Mei Su, Ying Hu, Jiaxin Sun, Wenzhong Chang, Yaru Li, Peijuan Tang, Yajuan Cui, Yujia Ma, Yanting Wang, Fengxian Zhang, Jia Wang, Yuchong Hu

**Affiliations:** ^1^Department of Gynaecology, Inner Mongolia People’s Hospital, Hohhot, China; ^2^Hospital Administration Office, Inner Mongolia People’s Hospital, Hohhot, China; ^3^Department of Clinical Medical Research Center, Affiliated Hospital of Inner Mongolia Medical University, Hohhot, China; ^4^Department of Nursing, Inner Mongolia People’s Hospital, Hohhot, China; ^5^Department of Nursing, Ordos Traditional Chinese Medicine Hospital, Ordos City, China; ^6^School of Nursing, Inner Mongolia Medical University, Hohhot, China; ^7^STD/AIDS Prevention and Control Section, Tongliao Center for Disease Control and Prevention, Tongliao, China; ^8^Maternity Department, Inner Mongolia People’s Hospital, Hohhot, China

**Keywords:** nursing interns, professional benefit perception, compassion satisfaction, secondary traumatic stress, mediation

## Abstract

**Aim:**

This study aims to examine the current professional benefit perception among nursing interns and explore their interactions with perspective taking, compassion satisfaction, and secondary traumatic stress.

**Background:**

The professional benefit perception is a crucial determinant in the career decisions of nursing interns. Understanding the pathways through which various factors influence this perception can inform the development of targeted intervention strategies. Such strategies are essential for preventing the attrition of nursing professionals and addressing the shortage of nursing human resources.

**Design:**

A cross-sectional design.

**Methods:**

Cross-sectional data were obtained from Inner Mongolia, China. To examine the influencing factors and pathways, multiple linear regression and the Hayes PROCESS macro were employed.

**Results:**

The study encompassed 427 nursing interns, whose perception of professional benefits was assessed at a moderate to high level, with a mean score of 4.29 ± 0.61. This perception was significantly affected by perspective taking (*t* = 3.990, *p* < 0.001), compassion satisfaction (*t* = 9.073, *p* < 0.001), secondary traumatic stress (*t* = −3.918, *p* < 0.001), overall satisfaction, and academic performance. Compassion satisfaction served as a mediator in the relationship between perspective taking and professional benefit perception, with a mediation effect value of 0.167, constituting 62.78% of the total effect. Furthermore, secondary traumatic stress moderated the relationship between compassion satisfaction and professional benefit perception, with an interaction effect value of *β* = 0.067 (*p* < 0.05).

**Conclusion:**

The professional benefit perception among nursing interns is shaped by a multitude of factors. Consequently, clinical educators should consider integrating these multidimensional factors to develop precise intervention programs aimed at enhancing professional identity and supporting the development of nursing talent.

## 1 Introduction

The global healthcare system is currently experiencing a significant shortage of nursing personnel, and the career stability of nursing interns has emerged as a crucial factor in addressing this talent deficit. Professional benefit perception, a core indicator of nursing staff’s perception of professional value, plays a pivotal role in influencing the career choices of nursing interns (1). This concept encompasses the comprehensive perception of growth experiences, social identity, and self-realization that practitioners acquire during clinical practice, with its formation process being integral to the dynamic evolution of professional cognitive reconstruction and identity. The internship phase serves as a critical transitional period for nursing students as they progress to becoming practicing nurses, and the development of their professional cognition during this time directly impacts their career decision-making post-graduation (2–4). Existing evidence (5, 6) indicates that a strong professional benefit perception significantly enhances the retention of nursing talent, whereas individuals with a diminished sense of benefits face a substantially higher risk of attrition (7). Nonetheless, the nursing trainee population currently exhibits a generally weak professional identity, with a notable underrepresentation of graduates from educational institutions, a trend that is especially evident during the middle and later stages of clinical practice (1, 8, 9).

The clinical period represents a pivotal phase for nursing interns to develop their professional cognitive reconstruction and identity (10). Existing research (11) indicates that this stage facilitates the transition from disciplinary knowledge to professional perception, achieved through the interplay of immersive clinical experiences and theoretical practice. Notably, the perception of professional benefit, as a central mediating variable, exerts a significant and positive influence on nurses’ sense of career belonging and their long-term practice intentions, mediated by the reinforcement of social value identity (12, 13). Additionally, demographic factors (such as age, years in nursing, and educational background) and occupational stressors have been shown to affect the development of benefit perception through their interactions, while psychological load may exert an indirect influence via the mediating role of personality traits (14, 15). Nonetheless, the majority of these studies have concentrated on practicing nurses, leaving the specific mechanisms underlying the formation of professional benefit perception among nursing interns insufficiently explored. Compared to experienced nurses, this cohort faces the dual challenges of acquiring clinical skills and adjusting psychologically to their evolving professional roles. Consequently, it is crucial to address nursing interns’ perceptions of professional benefits.

From the standpoint of individual development, a deficiency in perceived professional value can trigger a detrimental cycle of emotional exhaustion and burnout (16). In clinical practice settings, nursing interns frequently encounter the dual pressures of needing to rapidly enhance their technical skills while managing the empathic burden associated with patient suffering. When interns engage in perspective taking to actively construct cognitive frameworks, this altruistic practice may become a fundamental component of perceived professional value. According to the empathy-altruism hypothesis (17), individual variations in empathic ability can influence the development of professional value perceptions by affecting the transformative efficacy of altruistic motivation. It is important to note that the impact of empathy on the perception of occupational benefit is dual-faceted. Moderate empathic engagement can enhance career benefits through altruistic satisfaction, whereas excessive empathy, when experienced continuously in traumatic situations without psychological adjustment mechanisms, may lead to secondary traumatic stress (STS). Moreover, this adverse traumatic experience not only negates the positive outcomes of empathy but also diminishes career benefits through cognitive restructuring mechanisms (18).

At the health service system level, interns, as an essential reserve component of the nursing workforce, play a crucial role in shaping the future sustainability of nursing services. The quality of their professional benefit cultivation is directly linked to this sustainability. In the context of frequent public health emergencies, the high emotional demands characteristic of clinical internships complicate the interplay between empathic experiences and traumatic stress (19). Empathic energy can effectively contribute to a sense of professional fulfillment when it is channeled into an intrinsic motivation for professional commitment within appropriate boundaries. Conversely, if traumatic stress is not addressed promptly, it may undermine this sense of professional fulfillment and transform empathy into a detrimental cycle of self-depletion. The dynamic interplay between compassion satisfaction (CS) and STS may serve as a pivotal factor in determining whether individuals remain in or leave their careers under extreme stress.

This study aims to evaluate the current characteristics of nursing interns’ perceptions of professional benefits. Utilizing the empathy-altruism theory, it seeks to conduct an in-depth analysis of how viewpoint selection ability influences professional benefit perception through CS, while also examining the moderating effect of STS. The findings are expected to provide theoretical support for developing psychological intervention systems in clinical education, thereby effectively preventing the attrition of nursing talent and offering insights into mitigating the shortage of nursing human resources.

## 2 Background

The concept of “professional benefit perception” was initially introduced by Kramer (20), an American nursing scholar. Kramer highlighted that nurses derive positive emotional benefits from their professional practice, which include assisting others, achieving professional growth, and realizing social value. This concept encompasses not only the realization of self-worth and professional development within the profession but also enhances professional identity through social support and emotional satisfaction, thereby promoting professional stability and psychological well-being (21). Consequently, “professional benefit perception” is also referred to as “occupational value perception” (22).

Existing research indicates that the perception of professional benefits is linked to practitioners’ likelihood of engaging in occupational behaviors. A heightened sense of benefit can foster positive occupational behaviors while mitigating the risk of burnout (23). As the foundational cohort in the socialization of nursing careers, understanding the formation mechanism of nursing interns’ perception of occupational benefits is crucial. Presently, the study of professional benefit perception has broadened to encompass various healthcare domains, including professional groups such as nurses (21), physicians (24), and occupational therapists (25). It is noteworthy that nursing interns, serving as a strategic reserve of nursing human resources, possess a sense of career benefit that is intricately connected not only to their individual career commitment and professional development trajectory but also to the sustainable development of the nursing profession through talent retention mechanisms. A deficiency in career benefits may result in an elevated risk of burnout, increased intention to leave, and stagnation in career socialization (26).

Although there is a consensus on the significance of perceiving professional benefits, the underlying mechanisms remain inadequately understood. The empathy-altruism hypothesis offers a theoretical framework for examining this phenomenon. According to this hypothesis, individuals may engage in professional caregiving behaviors driven by altruistic motives when they exhibit empathetic attentiveness towards their clients (27). Batson’s theory of empathy further posits that cognitive empathy—defined as the capacity to accurately comprehend another person’s situation and needs through perspective-taking—is essential for the emergence of affective empathy (28). Perspective-taking, the cognitive foundation of empathy, involves the psychological process by which individuals transcend their self-centered viewpoints during interpersonal interactions to grasp others’ subjective experiences through cognitive restructuring. In the context of nursing practice, interns develop cognitive representations of patients’ experiences through perspective-taking. This involves not only identifying indicators of pain in patients’ nonverbal behaviors via clinical observation but also interpreting their emotional needs within the framework of their medical conditions. This cognitive process activates affective resonance responses in the anterior cingulate cortex and the mirror neuron system, facilitating interns to establish an emotional connection based on simulated patient experiences (29). When interns translate this cognitive-emotional integration into specific caring behaviors, the positive feedback from patients’ symptom improvement reinforces their perception of professional efficacy, ultimately affirming their professional value through the practice of altruistic behaviors.

Nevertheless, the occupational implications of empathic engagement are multifaceted. While sustained experiences of CS can enhance motivation to help, Stamm’s study (30) indicates a dual nature of empathic engagement among helpers. This engagement may either augment the perception of professional benefits via CS or incite STS due to excessive empathy. According to post-traumatic growth theory (31), the adverse effects of STS can potentially be mitigated or even transformed into opportunities for developing professional competence when helpers find meaning in their work. For nursing interns, encountering STS in clinical practice is unavoidable; however, the direction of its moderating effect—whether positive or negative—requires empirical investigation. The threshold effects and transformative mechanisms of such empathic engagement represent a crucial theoretical point for understanding the pathways through which a professional sense of benefit is formed.

**Figure 1:**
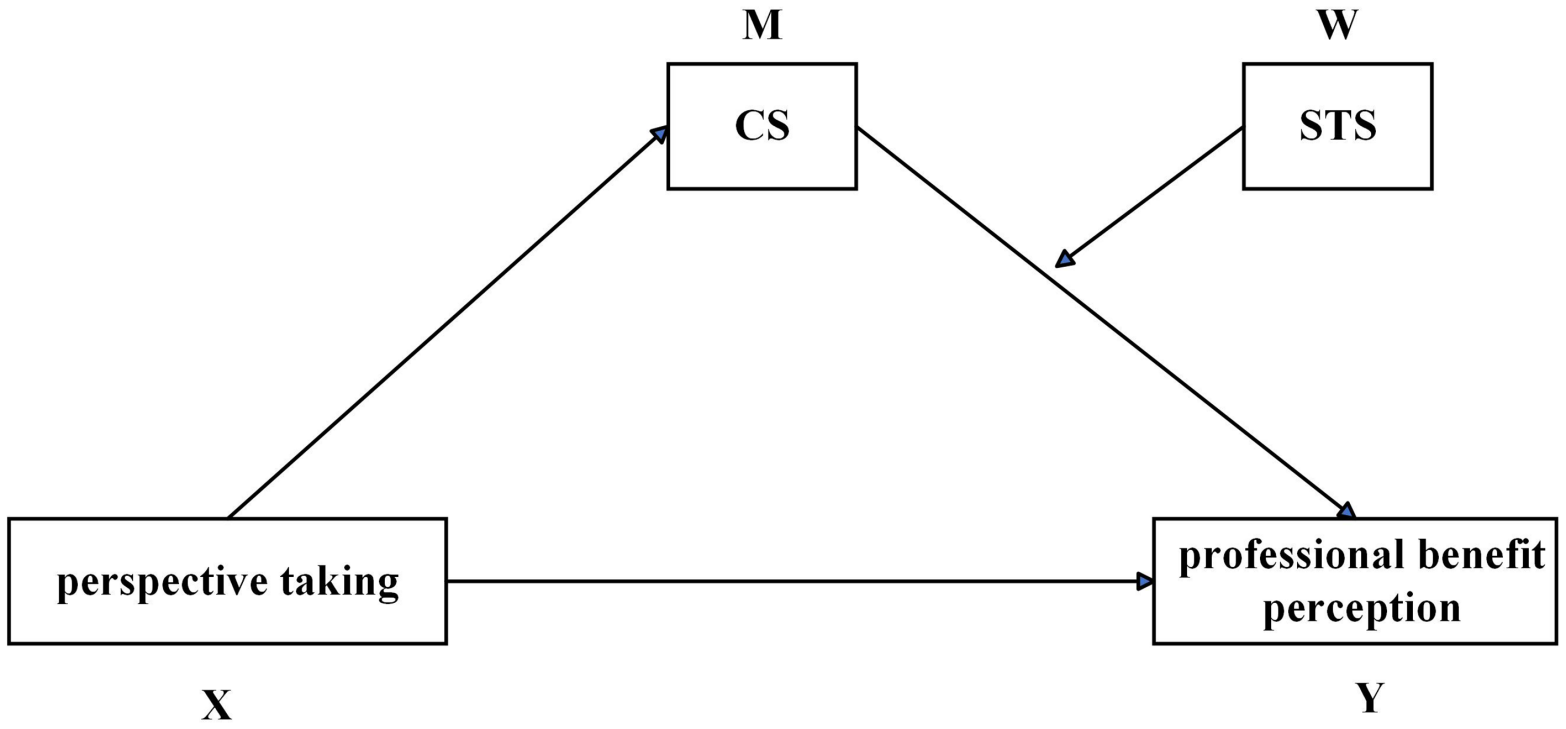
Conceptual framework diagram. This figure illustrates the moderated mediation model in which STS (W) moderates the effect of perspective taking (X) on professional benefit perception (Y) through CS (M).

Previous research has predominantly examined the impact of individual factors on nursing interns’ perceptions of professional benefits, leaving the mechanisms of multivariate interactions inadequately understood. This study aims to explore the following aspects: (1) the overall level of nursing interns’ perceptions of professional benefits; (2) the effects of perspective taking, CS, and STS on professional benefit perception; (3) and the interrelationships among perspective taking, CS, STS, and professional benefit perception. Specifically, this study establishes a conceptual model (Figure 1), with perspective taking (X) as the independent variable, professional benefit perception (Y) as the dependent variable, CS (M) as the mediator variable, and STS (W) as the moderator variable. This model posits that perspective taking influences professional benefit perception through CS (mediating pathway), while STS moderates the effect of perspective taking on CS (i.e., moderating the first stage of the mediation effect). The findings of this study are expected to provide a scientific foundation for optimizing clinical teaching strategies and developing a phased intervention program, which will be of significant practical value in mitigating interns’ professional identity crises and enhancing the stability of the nursing workforce.

## 3 Methods

### 3.1 Study design

This study is a cross-sectional study. Guidelines for the Strengthening the Reporting of Observational Studies in Epidemiology (STROBE) (Supplementary Material 1).

### 3.2 Participants and settings

This investigation was conducted as a multicenter cross-sectional study. A convenience sampling method was employed to select a cohort of 427 nursing interns who were engaged in practice from July 2024 to March 2025 across 10 tertiary general hospitals in Inner Mongolia. The inclusion criteria for participants were: (1) full-time nursing students enrolled in general higher medical education institutions nationwide; (2) currently undertaking a professional internship in the clinical departments of the hospitals, with a minimum internship duration of three months; and (3) provision of informed consent and voluntary participation in the study. Individuals who had not completed consecutive internships within the same hospital were excluded from the study. Sample size was determined using G Power 3.1 software. Parameters were configured as follows: Test family = *F* tests, Statistical test = Linear multiple regression: Fixed model, *R^2^* deviation from zero; Effect size *f^2^* = 0.15 (per Cohen’s medium effect size convention); *α* err prob. = 0.05 (two-tailed); Power (1-*β*) = 0.90; Number of predictors = 17. The calculation yielded a minimum required sample size of *N* = 179. Accounting for an anticipated 20% attrition rate, the adjusted sample size was approximately 215. In accordance with empirical guidelines for multiple linear regression analysis (requiring 10–20 samples per independent variable), 17 predictors necessitated 170–340 participants. Consequently, 427 nursing interns were ultimately enrolled.

The Inner Mongolia was stratified into three geographical regions—eastern, central, and western—comprising a total of 12 cities. For considerations of workload, cost, and suitability of the sample group, eight cities were selected based on the distribution of regional health resources: three from the eastern region, two from the western region, and three from the central region. Ten tertiary general hospitals were chosen as sample institutions, based on the probability proportional to the size of hospital internships. In conducting convenience sampling across ten hospitals, sampling quotas were initially distributed according to the total number of nursing interns present in each hospital, with quotas ranging from 35 to 50 cases per hospital. The hospital internship management departments facilitated the gathering of interns, while researchers collected data by distributing QR codes linked to electronic questionnaires to these departments. To ensure sample diversity, each hospital was mandated to include interns from a minimum of four clinical departments: internal medicine, surgery, emergency department, and operating room. Ultimately, the study sampled a total of 427 cases, surpassing the minimum required sample size of 204 cases. This excess was intended to mitigate the potential effects of non-response and missing data on the study’s outcomes. According to the sample size estimation formula, the minimum sample size requirement for this study was 204 cases. Therefore, the inclusion of 427 samples provides a robust representation of the nursing intern population across all 3A general hospitals in the Inner Mongolia.

### 3.3 Data collection

This study employs the Questionnaire Star online platformṅ^1^ for data collection and implements a comprehensive data quality control system to ensure the validity and reliability of the data. During the questionnaire design phase, a systematic literature review was conducted, and the initial questionnaire design and content were refined with input from experts in the field. In the distribution and collection phase, measures were taken to prevent duplicate responses, including restricting submissions to a single device IP address, enabling a WeChat account verification mechanism to detect and exclude repeated submissions, and setting a minimum completion time threshold of 90 s to automatically filter out invalid data from rapid and random responses. During the data processing phase, responses with mechanical answers, logical inconsistencies, errors in attention questions, and excessive missing values were eliminated. A total of 504 questionnaires were collected, and after applying quality control measures, 427 valid questionnaires were finally retained, with a valid questionnaire response rate of 84.7%.

### 3.4 Instruments

#### 3.4.1 Demographic characteristic

Following a comprehensive review of the literature, the research team developed and assembled a customized general information questionnaire. This instrument encompassed variables such as age, duration of internship, gender, only-child status, family circumstances, place of origin, academic performance, leadership roles, educational qualifications, whether the college attended was the first choice, part-time work experience, monthly disposable income, and overall satisfaction.

#### 3.4.2 Nurses’ professional benefit perception scale

For the assessment tool, the study employed the Professional Benefit Perception Scale, as developed by Hu (32), which comprises 33 items across five dimensions: self-growth, positive nurse–patient relationships, recognition from family and friends, positive career perception, and a sense of belonging to the team. Each item was evaluated using a 5-point Likert scale, ranging from “strongly disagree” (1 point) to “strongly agree” (5 points), resulting in a theoretical total score range of 33 to 165 points. Higher scores indicated a greater perceived occupational benefit for nursing interns. The scale demonstrated high internal consistency in this study, with an overall Cronbach’s alpha coefficient of 0.977, and the split-half reliability coefficient was 0.933, suggesting the scale’s stability in measurement within this study population. This instrument facilitates a systematic evaluation of nursing interns’ perceived professional values and developmental experiences.

#### 3.4.3 Jefferson scale of empathy

The scale, developed by Mohammadreza Hojat and his research team at Jefferson University in the United States, is primarily utilized to evaluate empathy levels among healthcare professionals. This instrument comprises 20 items categorized into three dimensions: perspective taking, affective care, and transpersonal thinking. Notably, the dimensions of affective care and transpersonal thinking employ a reverse scoring method. The scale is scored using a seven-point Likert scale, where forward items are rated from 1 (completely disagree) to 7 (completely agree), and reverse items are rated from 1 (completely agree) to 7 (completely disagree), with higher aggregate scores indicating greater empathy. In the present study, the scale demonstrated a Cronbach’s alpha coefficient of 0.910 and a split-half reliability of 0.882, reflecting good internal consistency reliability.

#### 3.4.4 Professional quality of life scale, ProQOL

The current study employed the internationally recognized Stamm (30) revised ProQOL Scale, a tool designed to assess professional quality of life across three distinct yet interrelated dimensions: compassion satisfaction (CS), burnout, and secondary traumatic stress (STS). Each dimension comprises 10 structured items, culminating in a total of 30 items for the entire scale. Responses were measured using a 5-point Likert scale, ranging from “never” (1 point) to “always” (5 points), with items 14, 15, 17, and 29 subjected to reverse scoring. The theoretical score range for each dimension spans from 10 to 50. This range reflects the positive psychological resources inherent in helping professions through the CS dimension, while the burnout and STS dimensions provide a systematic evaluation of the negative impacts associated with occupational stress. Higher scores indicate either an enhancement in positive satisfaction or an increase in psychological depletion as experienced by individuals engaged in empathetic occupational roles. The study confirmed the scale’s reliability, as evidenced by an overall Cronbach’s *α* coefficient of 0.925 and split-half reliability of 0.817. Dimension-specific reliability indices were as follows: CS (Cronbach’s α = 0.920, split-half = 0.896), burnout (Cronbach’s α = 0.750, split-half = 0.691), and STS (Cronbach’s α = 0.884, split-half = 0.826). These psychometric properties collectively demonstrate good internal consistency reliability, exceeding recommended thresholds for research applications.

### 3.5 Data analysis

Data processing and analysis were conducted using SPSS Statistics version 24.0 and Microsoft Excel. The normality of continuous variables was initially evaluated using the Kolmogorov–Smirnov test, supplemented by Q-Q plots and skewness/kurtosis indices. Categorical variables were presented as frequency (*n*) and composition ratios (%), while normally distributed continuous variables were expressed as mean ± standard deviation (*M* ± *SD*), and non-normally distributed continuous variables were reported as median [interquartile range] (M[Q1, Q3]). Statistical inference was rigorously aligned with the types and distribution properties of the variables. For group comparisons involving categorical variables, chi-square tests or Fisher’s exact tests were employed when expected frequencies were less than five. For continuous variables, independent t-tests were used for normally distributed data with two groups, and one-way ANOVA was applied for three or more groups. Non-normally distributed data were analyzed using Mann–Whitney U tests for two groups or Kruskal-Wallis H tests for three or more groups. Association analyses utilized Pearson correlation for normally distributed variables and Spearman’s rho for non-normally distributed variables. Multiple linear regression analysis controlled for multicollinearity by employing variance inflation factors (VIF < 5) and utilized stepwise variable selection. All constructs (perspective taking, CS, STS, professional benefit perception) were operationalized as observed variables using scale means. Hayes’ PROCESS macro (Models 4/14) tested mediation effects using bias-corrected bootstrapping (5,000 resamples) to generate 95% confidence intervals, a distribution-free approach requiring no normality assumptions. This study employed a systematic approach to address missing data, as outlined below: ① Data Screening and Classification: Initially, the missing data patterns across all variables were scrutinized to determine the nature of the missingness (MCAR, MAR, or MNAR) using Little’s MCAR test. Questionnaires with >20% missing variables were excluded per methodological standards. ② Single Variable Missingness Handling: For variables with <20% missing rate, Multiple Imputation (MI) was implemented via the mice package in R (v4.1.0) (33). Predictive models were built using predictive mean matching (PMM) with 20 imputed datasets, incorporating auxiliary variables to satisfy MAR assumptions. ③ Statistical Analysis Adjustment: During multiple linear regression, Robust Standard Errors (Huber-White estimators) were applied for bias-corrected inference (34). Bootstrap validation (5,000 resamples) (35) further confirmed model stability, with 95% CIs derived from bias-corrected accelerated intervals.

### 3.6 Ethical considerations

The studies involving human participants were reviewed and approved by the Ethics Committee of Inner Mongolia (approval no. 202509004L). All nursing interns who participated in this study were adults aged 18 or above. Prior to data collection, each participant provided written informed consent, confirming their voluntary participation and understanding of the study’s purpose, procedures, and potential risks. Since all participants were adults, there was no need for legal guardians or next of kin involvement in the consent process.

## 4 Results

### 4.1 Descriptive and univariate analysis

A total of 427 nursing interns participated in the study, with ages ranging from 20 to 24 years and a mean age of 21.43 years (SD = 1.16). The cohort comprised 65 male interns (15.2%) and 362 female interns (84.8%), including 145 undergraduate interns (34.0%) and 282 specialized interns (66.0%). The study findings indicated that academic performance, whether the college attended was the first choice, and overall satisfaction significantly influenced the interns’ perceived career benefits. As detailed in Table 1, these differences were statistically significant (*p* < 0.05). *Post hoc* multiple comparisons revealed that interns with the highest and middle academic performance reported a significantly greater sense of career benefit compared to those with lower performance. Additionally, the “very satisfied” group demonstrated higher scores than the “satisfied,” “average,” and “dissatisfied” groups, while the “satisfied” group exhibited a greater sense of career benefit than the “average” group (Supplementary Material 2).

**Table 1:** Comparison of professional benefit perception based on nursing interns’ demographic characteristic (*N* = 427).

Variables	*n* (%)	Mean ± SD, score	t/F	*P*
Gender				
Male	65 (15.2)	4.27 ± 0.71	−0.244	0.807
Female	362 (84.8)	4.29 ± 0.59		
Only-child status				
Yes	106 (24.8)	4.33 ± 0.62	0.744	0.457
No	321 (75.2)	4.28 ± 0.61		
Family circumstances				
Two-parent family	369 (86.4)	4.30 ± 0.60	0.512	0.600
Single-parent family	43 (10.1)	4.25 ± 0.66		
Remarried family	15 (3.5)	4.15 ± 0.67		
Place of origin				
Rural area	283 (66.3)	4.30 ± 0.62	0.665	0.515
Town	89 (20.8)	4.23 ± 0.58		
City	55 (12.9)	4.34 ± 0.59		
Academic performance				
Top 3 in the grade	28 (6.6)	4.43 ± 0.67	8.419	<0.001
Average	375 (87.8)	4.31 ± 0.58		
Below-average	24 (5.6)	3.82 ± 0.77		
Leadership roles				
Yes	117 (27.4)	4.37 ± 0.59	1.729	0.085
No	310 (72.6)	4.26 ± 0.61		
Educational qualifications				
Bachelor’s degree	145 (34.0)	4.30 ± 0.59	0.341	0.733
Associate degree	282 (66.0)	4.28 ± 0.62		
Whether the college attended was the first choice				
Yes	313 (73.3)	4.35 ± 0.60	3.777	<0.001
No	114 (26.7)	4.11 ± 0.62		
Part-time work experience				
None	137 (32.1)	4.24 ± 0.65	0.668	0.513
Occasionally	221 (51.8)	4.31 ± 0.59		
Frequent	69 (16.2)	4.31 ± 0.60		
Monthly disposable income				
≤2000 yuan	352 (82.4)	4.30 ± 0.60	2.362	0.053
2001 ~ 3,000 yuan	56 (13.1)	4.33 ± 0.60		
3,001 ~ 4,000 yuan	9 (2.1)	4.31 ± 0.65		
4,001 ~ 5,000 yuan	5 (1.2)	3.70 ± 0.93		
≥5,001 yuan	5 (1.2)	3.72 ± 1.00		
Overall satisfaction				
Very satisfied	109 (25.5)	4.62 ± 0.48	32.893	<0.001
Satisfied	196 (45.9)	4.33 ± 0.56		
Neutral	113 (26.5)	3.91 ± 0.58		
Dissatisfied	9 (2.1)	3.89 ± 0.74		

For a binary independent variable, use a t-test; for an independent variable with three or more categories, employ an F-test.

### 4.2 Current analysis of nursing interns’ professional benefit perception

Table 2 indicates that nursing interns generally experience a moderate to high professional benefit perception. A one-sample t-test revealed that the mean values for professional benefit perception (self-growth, positive nurse–patient relationships, recognition from family and friends, positive career perception, and a sense of belonging to the team), professional quality of life (CS and burnout), and empathy ability (perspective taking) were significantly above the theoretical median.

**Table 2:** Current situation analysis.

Dimensions	Score (mean, SD)	Test value	*t*
Total of professional benefit perception	4.29 ± 0.61	3.00	43.584^***^
Self-growth	4.38 ± 0.64	3.00	44.870^***^
Positive nurse–patient relationships	4.34 ± 0.63	3.00	44.111^***^
Recognition from family and friends	4.38 ± 0.63	3.00	45.250^***^
Positive career perception	4.11 ± 0.65	3.00	35.234^***^
A sense of belonging to the team	4.22 ± 0.63	3.00	40.179^***^
Total of ProQOL	3.18 ± 0.59	3.00	6.347^***^
CS	3.57 ± 0.76	3.00	15.701^***^
Burnout	3.22 ± 0.59	3.00	7.725^***^
STS	2.75 ± 0.78	3.00	−6.517^***^
Total of empathy	4.12 ± 0.70	4.00	3.488^***^
Perspective taking	5.14 ± 0.95	4.00	24.872^***^
Affective care	3.11 ± 1.15	4.00	−15.920^***^
Transpersonal thinking	3.05 ± 1.25	4.00	−15.707^***^

*** indicates *p* < 0.001; in the 5-point Likert scale, 3 represents the neutral point, therefore the test value is set at 3; in the 7-point Likert scale, 4 represents the neutral point, therefore the test value is set at 4.

### 4.3 Pearson correlation analysis

The results of the Pearson correlation analysis showed that perspective taking, CS, burnout, and professional benefit perception were positively correlated (*r* = 0.414, *p*<0.01; *r* = 0.655, *p*<0.01; *r* = 0.331, *p*<0.01); affective care, transpersonal thinking and professional benefit perception were all negatively correlated (r = −0.238, *p*<0.01; r = −0.211, *p*<0.01).

### 4.4 Multiple linear regression analysis

We conducted a linear regression analysis incorporating variables that demonstrated statistical significance (*p* < 0.05) in the univariate analysis. The independent variables included dimensions of empathy and professional quality of life, with age and duration of internship as control variables, while the dependent variable was the professional benefit perception. Categorical variables related to general information were treated with dummy variable assignment, as detailed in Supplementary Material 3. The regression analysis indicated a satisfactory model fit, with an adjusted *R*-square of 0.504. This suggests that the independent variables accounted for 50.4% of the variance in the dependent variable, thereby enhancing our understanding of the factors influencing the professional benefit perception. The analysis identified perspective taking, CS, and STS as significant determinants of nursing interns’ professional benefit perception (Table 3).

**Table 3:** Multiple linear regression of professional benefit perception.

Variables			Unstandardized coefficient B	Standardized coefficient β	t	*p*	VIF
(Constant)			98.150		7.035	<0.001	
Control variables	Age		−0.812	−0.047	−1.325	0.186	1.066
	Duration of internship		0.467	0.045	1.253	0.211	1.090
Independent variables	Overall satisfaction	Satisfied	−1.878	−0.046	−1.036	0.301	1.728
		Neutral	−5.895	−0.129	−2.594	0.010	2.130
		Dissatisfied	−2.789	−0.019	−0.015	0.607	1.145
		Very dissatisfied	−48.102	−0.116	−3.294	0.001	1.056
		Very satisfied	0				
	Academic performance	Average	−0.405	−0.007	−0.144	0.885	1.793
		Below-average	−8.987	−0.103	−2.207	0.028	1.864
		Top 3 in the grade	0				
	Perspective taking		0.329	0.155	3.990	<0.001	1.303
	Affective care		−0.161	−0.065	−1.205	0.229	2.476
	Transpersonal thinking		0.171	0.032	0.600	0.549	2.424
	CS		1.396	0.524	9.073	<0.001	2.858
	Burnout		0.410	0.120	1.659	0.098	4.474
	STS		−0.603	−0.235	−3.918	<0.001	3.076
Adjusted R^2^	0.504						
F	29.822						
*P*	<0.001						

The dependent variable was professional benefit perception.

### 4.5 Common method bias test

The Harman one-factor method was employed to assess the presence of common method bias. The analysis revealed the existence of 12 factors with eigenvalues exceeding 1, and the variance accounted for by the largest factor was 32.99%, which is below the 40% threshold. Consequently, there was no significant common method bias detected in this study.

### 4.6 Analysis of mediating effects

The mediating effects were analyzed using the Model 4 mediation model within the SPSS macro program to examine the role of nursing interns’ CS in the relationship between perspective taking and professional benefit perception. The analysis revealed a significant total effect of perspective taking on professional benefit perception (*β* = 0.266, *p* < 0.001, 95% CI [0.210, 0.322]), suggesting that increased perspective taking is associated with enhanced professional benefit perception. Even after controlling for the mediating variables, perspective taking maintained a significant positive effect on professional benefit perception (*β* = 0.099, *p* < 0.001, 95% *CI* [0.048, 0.150]), albeit with a reduced effect size, indicating partial mediation. The mediating effect was quantified at 0.167 (95% CI [0.128, 0.206]), constituting 62.78% of the total effect. Furthermore, using Model 14 in the SPSS macro program, the mediated effect with moderation was assessed. The results confirmed the validity of Model 14, indicating that the latter part of the mediation model was moderated. Specifically, the interaction between CS and STS was significant in predicting professional benefit perception (*β* = 0.067, *p*<0.05, 95% *CI* [0.001, 0.133]), implying that STS serves as a moderating factor. The detailed results are presented in Table 4.

**Table 4:** Testing the moderating effect of STS on the mediation model.

Variables	Model 1: professional benefit perception		Model 2: professional benefit perception	
	*β*	*t*	*β*	*t*
Constant	-	-	3.036	7.730
Perspective taking	0.099	3.840^***^	0.102	4.065^***^
CS	0.474	14.560^***^	0.338	3.470^***^
STS	-	-	−0.416	−2.977^**^
CS*STS	-	-	0.067	1.980^*^
*R^2^*	0.448		0.484	
*F*	171.767		98.844	

*P < 0.05, **P < 0.01, ***P < 0.001.

**Figure 2:**
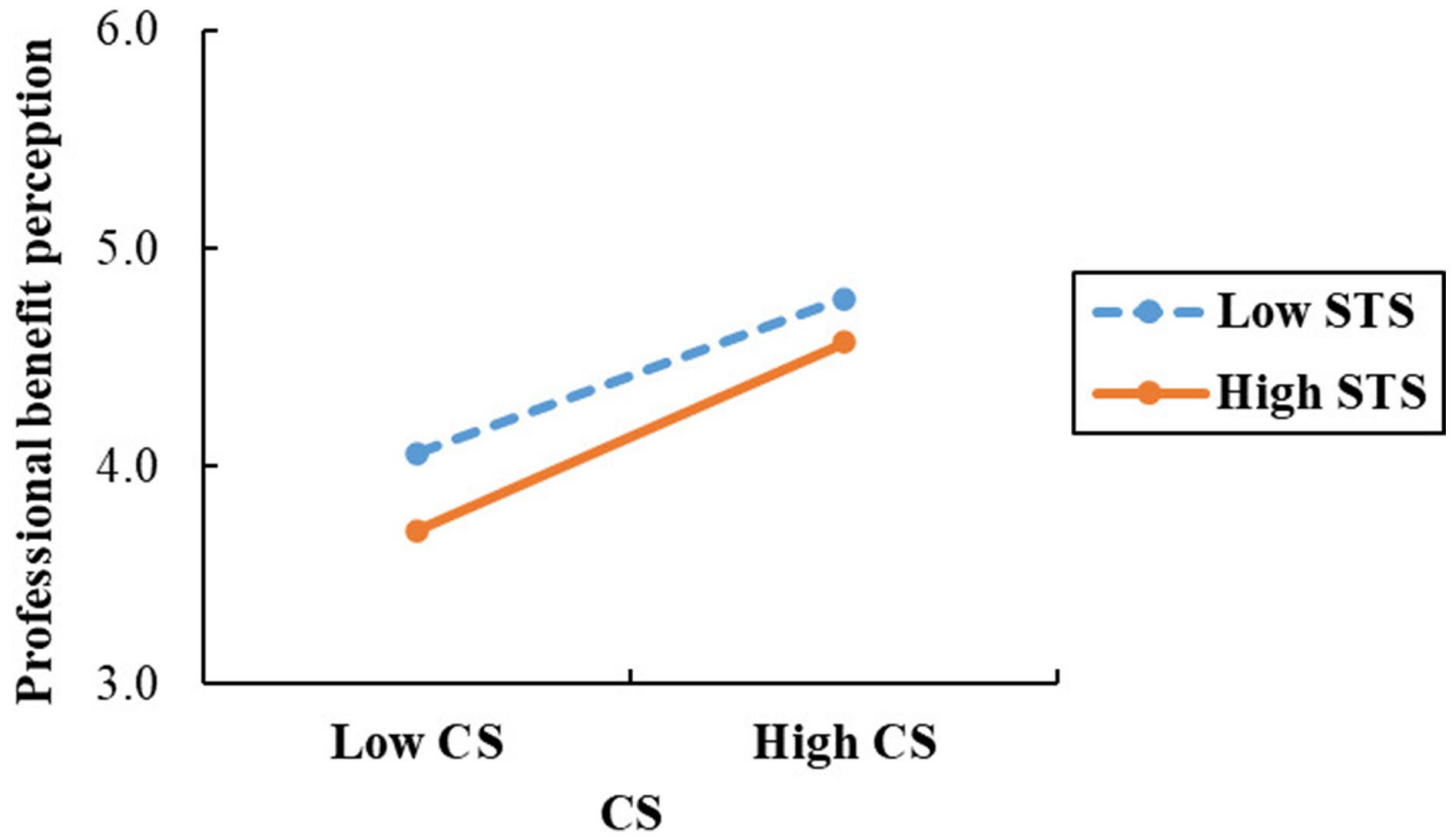
Simple slope plot. This figure showing how STS moderates the association between CS and professional benefit perception (higher STS strengthens the relationship).

A simple slope analysis further indicated that under high STS conditions (*M* + 1*S*), CS emerged as a significant positive predictor of perceived career benefits (*β* = 0.573, *t* = 13.429, *p* < 0.001, 95% CI [0.489, 0.657]). Similarly, in low STS conditions (*M*-1*S*), CS remained a significant predictor (*β* = 0.469, *t* = 11.313, *p* < 0.001, 95% *CI* [0.387, 0.550]), although the regression coefficient was reduced. This suggests that as the level of STS among nursing interns increases, CS continues to be a significant predictor of perceived professional benefits, with an enhanced positive effect. The findings are illustrated in Figure 2.

## 5 Discussion

The current study determined that nursing interns generally perceive their professional benefits as moderate to high, aligning with the results of previous research (36). In the dimensional analysis, the dimensions of self-growth and recognition from family and friends received high scores, which are closely associated with the characteristics of clinical practice. Nursing practice, as a critical component of knowledge translation, facilitates interns in gradually developing a professional identity through skill enhancement (37). Additionally, the experience of applying professional knowledge to provide health guidance to relatives and friends not only reinforces their sense of professional value but also boosts career satisfaction through positive feedback from relatives (38). Conversely, the dimension of positive career perception is relatively weaker, attributable to multiple factors. Nursing interns often encounter frustration due to the inadequate integration of theory and practice during the initial stages, compounded by the real pressures of graduate studies and employment, which can easily lead to STS (39, 40). Simultaneously, prevailing social stereotypes regarding the nursing profession, coupled with inadequate institutional support, have undermined the professional convictions of nursing interns (41). This complex issue indicates a need for a comprehensive enhancement of the nursing education system, focusing on three key areas: support for clinical practice, development of occupational psychology, and reshaping the professional image. Such improvements are essential to foster a sustained increase in nursing interns’ perceived career benefits.

Based on the findings of our study, the academic performance of nursing interns significantly influences their perception of professional benefits. This is particularly evident in the dual advantage observed among the top three grades and the middle-achieving group. Our results indicate that the knowledge and skills acquired during nursing education are not static; rather, they are progressively transformed into career development advantages through psychological resources such as career expectations and clinical adaptability (42). Notably, the exceptional performance of the middle-achieving group challenges the conventional belief in the “achievement-only theory.” Students who may not be at the top of their class but possess strong mental resilience can also build a professional identity through incremental successes in clinical practice. This gradual growth model offers a novel perspective on the cultivation of nursing talent (43). While existing research predominantly focuses on the career development trajectories of high-achieving students (44), our study reveals that middle-achieving students can also develop distinct career psychological advantages through effective feedback in clinical settings and role identity within teamwork. It is advisable to implement a “tiered empowerment” approach within educational practices, which would involve bolstering the professional leadership skills of students with exceptional academic performance and enhancing the ability of mid-performing students to apply their experiences through methods such as case reflection and clinical narrative. This approach ensures that students across various academic levels can identify pivotal points for their professional development.

The assertion that nursing interns’ overall satisfaction during their practicum significantly influences their perception of professional benefits has been corroborated by multiple studies (36, 45, 46). Various research findings indicate that fundamental components such as the practicum environment, faculty supervision, and the alignment of practicum content with clinical requirements directly impact interns’ perceived career benefits. From a practical standpoint, interns who report high levels of satisfaction exhibit enhanced clinical adaptability and achieve higher pass rates in skills assessments (47). This increased competence and positive career experience mutually reinforce each other, creating a virtuous cycle. When interns successfully perform independent nursing tasks under faculty supervision, they experience a heightened sense of career benefit. Clinical observations further suggest that nurses with a strong sense of career benefit achieve higher patient satisfaction scores and exhibit lower turnover rates (21). Satisfactory practicum experiences not only enhance the perceived value of the nursing profession but also effectively mitigate STS through improved competency and positive feedback (48). Consequently, the establishment of a supportive clinical education environment, the adoption of a mentorship teaching model, and the enhancement of occupational psychology intervention systems are crucial strategies to augment nursing interns’ sense of career benefit.

This study demonstrates a significant positive correlation between nursing interns’ ability to engage in perspective taking and their perception of professional benefits. This finding can be profoundly interpreted through the lens of the empathy-altruism hypothesis (27). According to Batson’s (27) theory, perspective taking, as the cognitive component of empathy, allows individuals to accurately discern others’ needs through transpositional thinking. In this study, interns with a high capacity for perspective taking were more proficient in identifying patients’ needs during clinical practice (49), thereby fostering a professional identity grounded in professional values by addressing these needs. This identity not only enhances their engagement in learning but also facilitates the attainment of career development goals by improving the autonomy of clinical decision-making (50, 51). Neuroscientific research provides biological evidence for this: Decety and Jackson (52) discovered, via functional magnetic resonance imaging, that the activation of the dorsal anterior cingulate gyrus and orbitofrontal cortex during perspective taking significantly overlaps with the neural activity regions associated with emotional empathy and decision-making related to helping behaviors. This suggests that when interns adopt the perspective of patients, they may evoke emotional resonance through the mirror neuron system, thereby activating the decision-making functions of the prefrontal cortex.

The interaction between cognition and emotion exerts varying effects on the perception of professional benefits throughout different stages of an internship. During the initial stages, novice interns, due to their limited clinical experience and professional knowledge, may find it challenging to translate their understanding of patients’ needs into a perception of professional benefit, despite possessing a high capacity for perspective-taking. This difficulty arises from the constraints imposed by their practical competence, which hinders the application of their cognitive insights. At this juncture, perspective-taking predominantly operates at a cognitive level, lacking substantial integration into clinical practice (53). Conversely, in the later stages of the internship, as interns acquire enhanced professional skills and accumulate clinical experience, they are better equipped to convert perspective-taking into practical altruistic actions. This capability allows them to derive professional value by effectively addressing patients’ needs, thereby markedly improving their perception of professional benefits (54). Furthermore, individual differences in personality traits may modulate this transformation process. For instance, interns with high levels of agreeableness may be more likely to translate perspective-taking into proactive professional behaviors, thereby enhancing their perception of professional benefits. Future research could further investigate this aspect.

CS plays a mediating role in this mechanism. Unlike mere emotional resonance, perspective-taking requires the cognitive subject to temporarily detach from an egocentric reference frame (55). This cognitive regulation capability enables interns to maintain emotional understanding of patients while avoiding emotional exhaustion from excessive involvement. Empirical studies have demonstrated that interns who effectively engage in perspective-taking report higher levels of clinical competence (56), suggesting that this ability may indirectly enhance the experience of professional benefits by improving intervention effectiveness. However, the simultaneous presence of STS in the scale indicates that clinical empathy involves more complex regulation (23, 57).

This study demonstrates that STS significantly moderates the relationship between CS and the perception of professional benefits in nursing clinical education. This phenomenon can be elucidated through the lens of post-traumatic growth theory. According to Tedeschi and Calhoun’s (68) framework, moderate exposure to trauma can catalyze cognitive restructuring and psychological growth in individuals. Although STS is typically perceived as a factor that diminishes CS (58), this study corroborates that during specific stages of clinical learning, particularly when STS levels reach a critical threshold, they substantially amplify the positive impact of CS on the perception of professional benefits (31). This effect may be attributed to the fact that, by the midpoint of their internships, interns have accrued sufficient clinical experience, making them more predisposed to engage in “cognitive restructuring” when confronted with traumatic events. This process enables them to transform negative experiences into opportunities for reconstructing their professional identity (59). For instance, following emotionally distressing events such as patient death, interns may reassess the value and mission of nursing, actively seek support from supervisors, modify their strategies for empathic expression, and elevate their empathic behaviors from passive emotional responses to the proactive practice of their professional mission, thereby significantly enhancing the depth of professional benefit perception.

The mechanism of transformation, however, exhibits considerable variability among individuals. Resilience levels play a crucial role in moderating the impact of STS; individuals with high resilience are more likely to engage the “personal strength” dimension of post-traumatic growth when confronted with STS, thereby transforming stress into a motivational force for growth. Conversely, individuals with low resilience may resort to avoidance coping strategies in response to persistent stress, thereby diminishing the positive influence of CS on their perception of professional benefits (60). Furthermore, psychological preparedness and the ability to access resources at various stages of internships influence the trajectory of post-traumatic growth. Longitudinal research conducted during the COVID-19 pandemic indicates that nursing interns with high resilience were more adept at maintaining mental health during clinical exposure and converting professional stress into growth motivation. In contrast, those with low resilience exhibited elevated symptoms of post-traumatic stress (61). This phenomenon may be attributed to mid-internship individuals having acquired sufficient clinical experience, thereby facilitating the activation of the “cognitive restructuring” mechanism more effectively. Future research could further introduce longitudinal tracking designs, integrate personality traits and social support variables, and construct more comprehensive theoretical models to provide targeted strategies for balancing emotional protection and professional identity cultivation in nursing education.

### 5.1 Implications for practice

① Enhance Perspective Taking Skills: Integrate standardized patient (SP) simulation training into clinical communication courses to improve professional benefit perception by enhancing CS. ② Develop a Dynamic Monitoring Mechanism for STS: Utilize Figley’s theoretical framework and employ the “Secondary Traumatic Stress Scale (STSS)” as the principal assessment tool. Conduct quarterly online evaluations for nursing interns and implement stress transformation interventions for high-risk groups. ③ Implement Specialized Empathy Management Training and Establish a “Stress-Growth” Transformation Support System: Offer targeted empathy management training for interns susceptible to STS and create a support system focused on transforming stress into growth. Refer to Table 5 for further details.

**Table 5:** Summarizes intervention measures for nursing interns.

Intervention measures	Target outcomes	Implementation strategies	Potential barriers	Overcoming strategies
Standardized Patient Simulation Training (62–64)	Improve students’ scores in doctor-patient communication skills and increase their scores on the empathy scale	Four simulation trainings per semester + reflection journals + peer evaluation	High labor cost of standardized patients	Train senior students to be SPs; Develop VR simulation systems
STS Dynamic Monitoring and MBSR Intervention (65, 66)	Reduce STSS scores among high-risk groups and decrease the incidence of occupational burnout	Quarterly evaluations + online mindfulness courses + offline group counseling	Low student participation rate	Include in internship assessment indicators; Provide credits for psychological interventions
Special Training on Empathy Management (67)	Ensure most students acquire fundamental empathy skills and enhance their satisfaction with clinical internships	6-week phased training + mentor supervision + online support platform	Time and resource conflicts	Adopt fragmented learning mode; Integrate existing teaching resources

### 5.2 Relevance to clinical practice

The roles of compassion satisfaction and secondary traumatic stress as mediating and moderating factors, respectively, in the development of professional benefit perception underscore the need for clinical education to focus on fostering empathy and managing trauma. This approach can enhance occupational identity through targeted interventions and optimize the nursing workforce.

### 5.3 Limitations

This study has several limitations. Firstly, its cross-sectional design hinders the establishment of causal time-series relationships between variables, necessitating future longitudinal studies to verify the dynamic causal pathways among perspective taking, compassion satisfaction, and perception of professional benefits. Additionally, this study is constrained by its reliance on sample recruitment from tertiary general hospitals in Inner Mongolia, which does not adequately represent the diversity of nursing education systems and internship models across various regions in China. In the eastern coastal areas, nursing education emphasizes interdisciplinary practice and personalized teaching, utilizing high-quality medical resources. In contrast, institutions in the central and western regions predominantly adhere to traditional, standardized theoretical instruction. Internships in primary healthcare facilities encounter challenges such as resource shortages and insufficient multidisciplinary collaboration, which markedly differ from the centralized and standardized training models prevalent in tertiary hospitals. Future research should broaden the scope of the sample to include nursing interns from both eastern and western regions, as well as from both tertiary and primary healthcare institutions, to facilitate a comparative analysis of the formation mechanisms of professional benefit perception under varying educational resource configurations. Additionally, to enhance the practical value of research findings, it is important to assess the applicability of the ‘compassion cultivation-stress transformation’ dual-track system in primary healthcare settings and optimize the threshold identification tools and intervention strategies for secondary traumatic stress.

## 6 Conclusion

This study demonstrates that nursing interns’ perceptions of professional benefits range from intermediate to high, influenced by a synergistic interplay of multidimensional factors, including perspective taking, CS, and STS. CS serves a pivotal role in mediating the relationship between perspective taking and professional benefit perception. Meanwhile, STS exerts a significant moderating effect, enhancing the predictive power of CS on professional benefit perception when certain thresholds are surpassed. The findings suggest that clinical education should implement a dual-track intervention system focused on “empathy cultivation and stress transformation.” Such an approach can effectively bolster nursing interns’ professional identity and provide strategic support for stabilizing nursing human resources by accurately identifying secondary factors.

## Data Availability

The datasets presented in this study can be found in online repositories. The names of the repository/repositories and accession number(s) can be found below: the data that support the findings of this study are available from the corresponding authors upon reasonable request.

## Footnotes

^1^ https://www.wjx.cn/
