# Supplementary material for: Pathway analysis of nursing interns’ professional benefit perception and influencing factors: a cross-sectional study
Source: Front Med (Lausanne). 2025 Aug 13;12:1628232. doi: 10.3389/fmed.2025.1628232 (PMC12380669; doi:10.3389/fmed.2025.1628232)
Supplement: Supplementary file 2 [file Table_2.docx]

*Supplementary Material 2*

Descriptive and univariate analysis

1 The results of the Bonferroni correction showed that the perceived occupational benefits of the below-average interns were significantly lower than those of the top 3 interns and the average trainees. (Table 1, Figure 1)

Table 1 Bonferroni correction

| I | J | Mean difference (I-J) | SE | P | 95% confidence interval | |
| --- | --- | --- | --- | --- | --- | --- |
|  |  |  |  |  | Lower limit | Upper limit |
| Below-average | Top 3 in the grade | -0.61508* | 0.16698 | 0.001 | -1.0164 | -0.2137 |
| Below-average | Average | -0.49182* | 0.12639 | 0.000 | -0.7956 | -0.1880 |


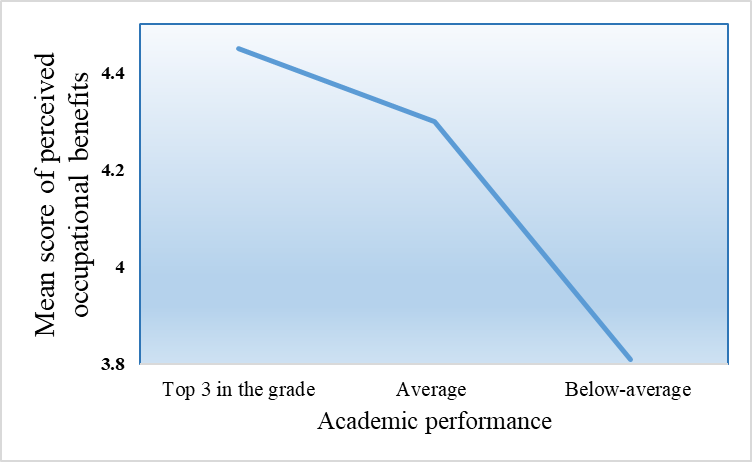


Figure 1

2 The results of Bonferroni correction showed that very satisfied interns had significantly higher perceived occupational benefits than satisfied, neutral and dissatisfied interns, and satisfied interns had significantly higher perceived occupational benefits than neutral satisfied interns (Table 2, Figure 2)

Table 2 Bonferroni correction

| I | J | Mean difference (I-J) | SE | P | 95% confidence interval | |
| --- | --- | --- | --- | --- | --- | --- |
|  |  |  |  |  | Lower limit | Upper limit |
| Very satisfied | Satisfied | 0.29055* | 0.06593 | 0.000 | 0.1158 | 0.4653 |
| Very satisfied | Neutral | 0.71105* | 0.07408 | 0.000 | 0.5147 | 0.9074 |
| Very satisfied | Dissatisfied | 0.73608* | 0.19138 | 0.001 | 0.2288 | 1.2434 |
| Satisfied | Neutral | 0.42050* | 0.06518 | 0.000 | 0.2477 | 0.5933 |


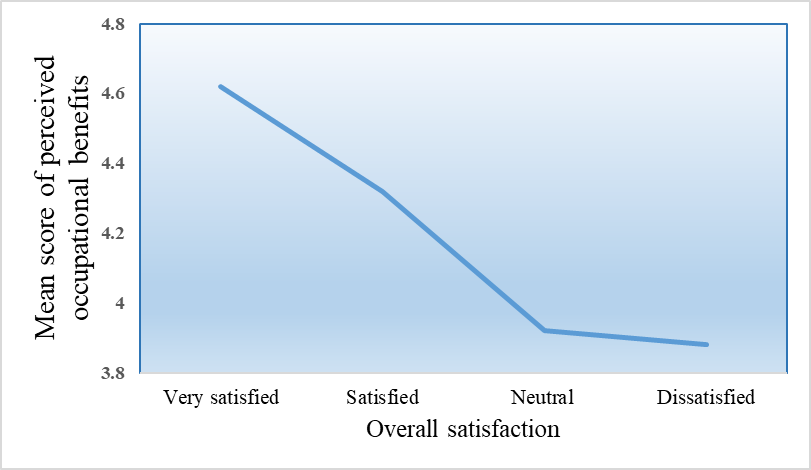


Figure 2
