# Supplementary material for: Pathway analysis of nursing interns’ professional benefit perception and influencing factors: a cross-sectional study
Source: Front Med (Lausanne). 2025 Aug 13;12:1628232. doi: 10.3389/fmed.2025.1628232 (PMC12380669; doi:10.3389/fmed.2025.1628232)
Supplement: Supplementary file 3 [file Table_3.docx]

*Supplementary Material 3*

Dummy Variable Coding

**Gender**

With "Male" as the reference category, "Female" is assigned the code "01".

**Place of Origin**

With "Rural area " as the reference category, "Town" is assigned the code "010", "City" is assigned the code "001".

**Monthly Disposable Income**

With "≤2000 yuan" as the reference category, "2001–3000 yuan " is assigned the code "01000", "3001–4000 yuan " is assigned the code "00100", "4001–5000 yuan " is assigned the code "00010", "≥5001 yuan " is assigned the code "00001".

**Educational Qualifications**

With "Bachelor’s Degree" as the reference category, "Associate Degree" is assigned the code "01".

**Part-time Work Experience**

With "None" as the reference category, "Occasionally" is assigned the code "010", "Frequent" is assigned the code "001".

**Overall Satisfaction**

With "Very Satisfied" as the reference category, "Satisfied" is assigned the code "01000", "Neutral" is assigned the code "00100", "Dissatisfied" is assigned the code "00010", "Very Dissatisfied" is assigned the code "00001".
